# Risk modifiers of immune-related adverse events in patients with NSCLC undergoing immunotherapy: a retrospective cohort study

**DOI:** 10.1186/s40780-026-00604-z

**Published:** 2026-06-27

**Authors:** Takashi Watanabe, Naoto Suzuki, Masahito Yamauchi, Naoki Iinuma, Makoto Shiozawa, Yotaro Arima, Takanobu Sasaki, Hideki Shimodaira, Syu-ichi Kanno, Kouji Okada

**Affiliations:** 1 Division of Clinical Pharmacotherapeutics, Tohoku Medical and Pharmaceutical University, 4-4-1, Komatsushima, Aoba-ku, Sendai, 981-8558 Japan; 2 Department of Pharmacy, Tohoku Medical and Pharmaceutical University Hospital, Sendai, 983-8512 Japan; 3 Department of Pharmacy, Tohoku Medical and Pharmaceutical University Wakabayashi Hospital, Sendai, 984-8560 Japan; 4 Department of Thoracic Surgery Tohoku Medical and Pharmaceutical University Hospital, Sendai, 983-8512 Japan; 5 Department of Clinical Oncology, Tohoku Medical and Pharmaceutical University Hospital, Sendai, 983-8512 Japan; 6 Division of Clinical Pharmaceutics and Pharmacy Practice, Tohoku Medical and Pharmaceutical University, Sendai, 983-8536 Japan

**Keywords:** Retrospective cohort study, Immune checkpoint inhibitors, Immune-related adverse events, Comorbid heart failure, Non-small cell lung cancer

## Abstract

**Background:**

To investigate immune-related adverse events (irAEs) in patients with non-small cell lung cancer receiving immune checkpoint inhibitor (ICI) therapy, and to identify clinical factors associated with irAE onset, thus supporting safer immunotherapy through effective monitoring.

**Methods:**

This retrospective cohort study was conducted at Tohoku Medical and Pharmaceutical University Hospital over approximately 5 years. Hospitalized patients with non-small cell lung cancer who received immune checkpoint inhibitors for the first time were included. Physicians and pharmacists evaluated the occurrence, severity, and timing of irAEs. Cox proportional hazards regression and Fine–Gray competing risk analyses were performed to identify factors associated with irAE development.

**Results:**

Among the 203 enrolled patients, 92 (45.3%) experienced irAEs, totaling 110 events. The most common irAE was interstitial lung disease (23 cases, 20.9%), followed by thyroid dysfunction (20 cases, 18.2%). In multivariable Cox regression analysis, comorbid heart failure was significantly associated with a reduced risk of irAE development (hazard ratio, 0.299; 95% confidence interval [CI], 0.135–0.663; *p* = 0.003), and this association remained significant in the Fine–Gray competing risk model (subdistribution hazard ratio, 0.335; 95% CI, 0.147–0.766; *p* = 0.009). To address potential multicollinearity, an alternative model incorporating regular use of renin–angiotensin–aldosterone system inhibitors in place of comorbid heart failure also demonstrated a significant association with irAE incidence.

**Conclusions:**

In this Japanese non-small cell lung cancer cohort, comorbid heart failure emerged as a potential modifier of irAE incidence, suggesting that baseline cardiac conditions may influence susceptibility to immune‑related toxicity. These findings underscore the importance of incorporating comorbidity profiles into individualized irAE monitoring strategies during cancer immunotherapy.

## Background

Non-small cell lung cancer (NSCLC) is a highly prevalent malignancy associated with significant mortality and limited responsiveness to conventional cytotoxic therapies [1, 2]. Recently, chemotherapy regimens incorporating immune checkpoint inhibitors (ICIs) have emerged as standard treatment options for patients with advanced or recurrent NSCLC and are now widely used either as monotherapy or in combination with other anticancer agents [3–5]. ICIs have been approved as first-line monotherapy for advanced NSCLC with programmed death-ligand 1 (PD-L1) expression ≥ 50% and are approved for use in combination with chemotherapy regardless of PD-L1 expression status. Evidence from randomized clinical trials has demonstrated that ICIs confer superior survival outcomes compared to conventional cytotoxic agents, highlighting their efficacy in the management of NSCLC [6].

ICIs exert their therapeutic effect by disrupting inhibitory signals transmitted from neoplastic cells to immune effector cells, thereby enabling activated T cells to target malignant cells. However, such immune activation may also trigger unintended T cell-mediated responses against self-antigens, resulting in a distinct spectrum of toxicities known as immune-related adverse events (irAEs). ICIs induce irAEs across multiple organ systems, including the dermatologic, gastrointestinal, pulmonary, endocrine, and cardiovascular systems [7, 8]. These adverse events vary in severity, ranging from mild to life-threatening; severe irAEs may necessitate treatment discontinuation and initiation of immunosuppressive therapy [9]. Notably, the onset of irAEs may be delayed by several weeks or months, highlighting the need for continuous and vigilant monitoring throughout ICI administration [10]. Recognizing the unique and potentially fatal toxicological profiles of ICIs is essential for the safe delivery of cancer immunotherapy.

Appropriate management of irAEs is essential for optimizing treatment outcomes and maintaining patients’ quality of life, while identifying factors associated with their onset remains a critical clinical priority. Several studies have investigated risk factors and potential biomarkers for irAEs in the context of cancer immunotherapy [11]. Zhang et al. emphasized the importance of integrating symptom management into the continuum of care for patients receiving ICIs, particularly among those with elevated body mass index (BMI) [12]. In patients with NSCLC, clinical variables, such as the use of programmed cell death protein 1 (PD-1) inhibitors (particularly pembrolizumab), combination with chemotherapy, smoking status, C-reactive protein levels, treatment response, and serum albumin levels are reported risk factors for the development of irAEs [13–15]. Fujimoto et al. reported that a low pretreatment neutrophil-to-lymphocyte ratio may serve as a predictive marker for irAEs in patients with NSCLC [16]. Nevertheless, inconsistencies across cancer types, ICI treatment regimens, patient demographics, and evaluation parameters persist, and no definitive predictive factors have been established.

Given these uncertainties, further clarification of the clinical factors associated with irAE development is warranted. Accordingly, in this retrospective cohort study, we aimed to characterize the spectrum of irAEs in patients with NSCLC receiving ICIs and to identify clinical factors associated with their occurrence, thereby supporting the implementation of comprehensive monitoring strategies for safer and more effective cancer immunotherapy.

## Methods

### Study design and population

Adult patients with NSCLC, either with postoperative recurrence or unresectable advanced cancer, who received ICI therapy for the first time at Tohoku Medical and Pharmaceutical University Hospital in Japan, were enrolled in this retrospective cohort study. Patients were identified as having received ICI therapy if they underwent at least one cycle of nivolumab, pembrolizumab, atezolizumab, or durvalumab between February 1, 2017, and December 31, 2021, administered either as monotherapy or combined with chemotherapy using anticancer agents such as pemetrexed, paclitaxel, or carboplatin. Data were reviewed from the initiation of ICI therapy to rigorously assess the presence or absence of irAEs during the treatment course. Irrespective of whether irAEs occurred during treatment, patients were systematically followed from the date of the final ICI administration until the last follow‑up, a predefined period after ICI discontinuation, or death, whichever occurred first. Patients who did not develop irAE were subsequently categorized according to disease progression, ICI discontinuation, or death.

### Definitions

The occurrence of irAEs was determined by physicians according to criteria described in previous reports [17, 18]. Severity was graded using the Common Terminology Criteria for Adverse Events (CTCAE) version 5.0 (Grades 1–5) and was evaluated jointly by physicians and pharmacists. Time to irAE onset was defined as the interval between the initial administration and the date on which the physician identified the irAE. The identified irAEs included interstitial lung disease (ILD), endocrine disorders (e.g., thyroid dysfunction, adrenal cortical dysfunction, pituitary dysfunction, and type 1 diabetes mellitus), colitis and/or severe diarrhea, dermatologic disorders, hepatic dysfunction, neurologic and muscular disorders, and renal dysfunction. Patients who developed immune-related fever, hypercalcemia suggestive of hyperparathyroidism, or oral mucositis were assessed by physicians as having irAEs and were categorized as other irAEs.

### Data collection

We characterized the patient population by extracting clinical data from electronic medical records of individuals who underwent cancer immunotherapy. The collected variables included age, sex, BMI, treatment indication (postoperative recurrence or advanced cancer), ICI-related characteristics, such as types of ICI (nivolumab, pembrolizumab, atezolizumab, or durvalumab), checkpoint properties (PD-1 antibodies or PD-L1 antibodies), ICI treatment regimens (monotherapy or combination with chemotherapy), and history of prior administration of anticancer agents other than ICIs. Additional variables were collected for the comparison between patients with and without irAEs. These included histological subtype (adenocarcinoma, squamous cell carcinoma, large cell carcinoma, or other/not otherwise specified); the Brinkman index (threshold of 600); comorbidities such as diabetes mellitus, heart failure, obstructive ventilatory disorders, and autoimmune diseases; regular use of strong opioids, oral corticosteroids, and inhaled corticosteroids, and renin–angiotensin–aldosterone system (RAAS) inhibitors; laboratory values obtained near the initiation of ICI therapy (aspartate aminotransferase, alanine aminotransferase, and serum creatinine); C-reactive protein levels measured 1–2 weeks after the first ICI administration, and the total number of ICI administrations. The Brinkman index was calculated as the product of the number of cigarettes smoked per day and the number of years of smoking, and patients were classified using a cutoff value of 600, which corresponds to heavy smoking [19, 20]. Patients were classified as having a comorbidity if the disease was explicitly documented in the electronic medical record based on a physician’s diagnosis and pharmacologic treatment for that disease ongoing at the time of the initial ICI administration. Patients who did not meet these criteria were classified as not having a comorbidity. To illustrate, heart failure was considered a comorbidity if it was explicitly documented in the electronic medical record and the patient was receiving pharmacological therapy for heart failure, such as RAAS inhibitors, β-adrenergic receptor blockers, diuretics, or digitalis, during initial ICI administration. Diabetes mellitus, obstructive ventilatory disorders, and autoimmune diseases were likewise defined as comorbidities using the same criteria.

### Statistical analysis

Univariate analysis was performed to compare variables between patients with and without irAEs. The Mann–Whitney *U* test was used for discrete and continuous variables, while Pearson’s chi-squared or Fisher’s exact test was applied for categorical variables, as appropriate. Cause‑specific hazard ratios (HRs) were estimated using Cox proportional hazards regression with the Wald test, with patients who did not develop irAEs censored at the last follow‑up or at a predefined time point after ICI discontinuation. Cumulative incidence functions were compared using Gray’s test, and subdistribution hazard ratios (SHRs) were estimated using the Fine–Gray competing risk model with the Wald test, in which competing events were incorporated into the subdistribution hazard. A p-value of < 0.05 was considered statistically significant.

### Ethics approval

The ethics committee of the Tohoku Medical and Pharmaceutical University Hospital approved the study (No. 2022-2-063-2024). We posted information about this study on the hospital website and allowed participants to opt out; those who did not opt out were considered to have provided tacit consent for study participation. This work was carried out in accordance with the Code of Ethics of the World Medical Association (Declaration of Helsinki) for experiments involving humans.

## Results

### Study patients

ICI-based immunotherapy was initiated in 237 patients for the treatment of NSCLC. After excluding 34 patients with incomplete survey data, 203 patients were included in the final analysis. Patient characteristics are summarized in Table 1. The median age was 71 years, and 151 patients (74.4%) were men. ICIs were first administered in 88 patients (43.3%) for postoperative recurrence and in 115 patients (56.7%) for advanced disease. The most commonly used ICI was pembrolizumab. ICI monotherapy was administered to 144 patients (70.9%), whereas ICI in combination with chemotherapy was administered to 59 patients (29.1%). Sixty-seven patients (33.0%) had previously received anticancer agents other than ICIs for NSCLC. Overall, 92 patients (45.3%) experienced irAEs.

**Table 1:** Patient population (*N* = 203)

	*N* (%) or Median [IQR]
Age (years)	71 [65-76.5]
Sex	
Men	151 (74.4)
Women	52 (25.6)
BMI	21.6 [19.4–24.7]
Treatment indication	
Postoperative recurrence	88 (43.3)
Advanced cancer	115 (56.7)
ICI-related characteristics	
Types of ICI	
Nivolumab	38 (18.7)
Pembrolizumab	120 (59.1)
Atezolizumab	34 (16.8)
Durvalumab	11 (5.4)
Checkpoint properties	
PD-1 antibodies	158 (77.8)
PD-L1 antibodies	45 (22.2)
ICI treatment regimens	
Monotherapy	144 (70.9)
Combination with chemotherapy	59 (29.1)
History of prior administration of anticancer agents	
Yes	67 (33.0)
No	136 (67.0)
Occurrence of irAEs	
Yes	92 (45.3)
No	111 (54.7)

*N* number, *IQR* interquartile range, *BMI* body mass index, *ICI* immune checkpoint inhibitor, *PD-1* programmed cell death protein 1, *PD-L1* programmed death-ligand 1, *irAEs* immune-related adverse events

### Types, severity, and onset timing of irAEs

Table 2 summarizes the types and severity of irAEs observed, with 110 events occurring in 92 patients. The most frequently reported irAE was ILD, observed in 23 patients (20.9%), followed by thyroid dysfunction in 20 patients (18.2%) and colitis or severe diarrhea in 17 patients (15.5%). The median time to irAE onset was 72 d (interquartile range [IQR], 23–184). Dermatologic disorders showed the earliest onset with a median of 17 d (IQR, 8–100), whereas neurologic and muscular disorders had the latest onset at a median of 189 d (IQR, 112–329). CTCAE Grade II irAEs were the most common, accounting for 57 events (51.8%). CTCAE Grade III irAEs were more frequently associated with ILD, whereas CTCAE Grade II predominated in thyroid dysfunction and colitis and/or severe diarrhea. This study documented one fatal case each of ILD and hepatic dysfunction, both assessed as CTCAE Grade V, in which a causal relationship with irAEs could not be excluded.

**Table 2:** Types, severity, and onset timing of irAEs (*n*: 110)

	*n* (%)	Onset timing	CTCAE grade (%)				
		Median days [IQR]	I	II	III	IV	V
ILD	23 (20.9)	70 [48–131]	2	5	15		1
Endocrine disorders	30 (27.3)	119 [56–234]					
Thyroid dysfunction	20 (18.2)	88 [43–154]	5	15			
Adrenal cortical dysfunction	6 (5.5)	156 [62–636]	1	4	1		
Pituitary dysfunction	3 (2.7)	153 [150–171]	2	1			
Type 1 diabetes mellitus	1 (0.9)	274			1		
Colitis and/or severe diarrhea	17 (15.5)	98 [59–234]	2	10	5		
Dermatologic disorders	14 (12.7)	17 [8-100]	5	9			
Hepatic dysfunction	9 (8.2)	99 [21–114]	1	5	2		1
Neurologic and muscular disorders	5 (4.5)	189 [112–329]	1	3	1		
Renal dysfunction	1 (0.9)	27			1		
Others	11 (10.0)		4	5	1	1	
Non-ILD	87 (79.1)	73 [22–193]	21	52	12	1	1
Total	110	72 [23–184]	23 (20.9)	57 (51.8)	27 (24.6)	1 (0.9)	2 (1.8)

Others: immune-related fever, hypercalcemia suggestive of hyperparathyroidism, or oral mucositis were assessed by the physician as having irAEs. *irAEs*, immune-related adverse events; n, number; *IQR*, interquartile ranges; *CTCAE*, Common Terminology Criteria for Adverse Events; *ILD*, interstitial lung disease

### Patient characteristics in the two groups

Table 3 summarizes the clinical characteristics of patients with and without irAEs. The proportion of patients with a Brinkman index ≥ 600 was significantly higher in the irAE group than in the non-irAE group (76.1% vs. 61.3%; *p* = 0.036). In contrast, comorbid heart failure and regular use of RAAS inhibitors were significantly more prevalent in the non-irAE group than in the irAE group (24.3% vs. 7.6% and 39.6% vs. 21.7%; *p* = 0.003 and *p* = 0.010, respectively). No other clinical parameters showed significant differences between the groups.

**Table 3:** Comparison of groups with and without irAEs (*N* = 203)

	Group with irAEs (*N* = 92)	Group without irAEs (*N* = 111)	*p*-value
Age (years)	70 [62–77]	71 [66.5–76]	0.201^a)^
Sex			0.730^b)^
Men	70 (76.1)	81 (73.0)	
Women	22 (23.9)	30 (27.0)	
BMI	21.5 [19.7–24.2]	21.7 [19.2–24.8]	0.731^a)^
Histological subtype			0.602^c)^
Adenocarcinoma	50 (54.3)	65 (58.6)	
Squamous cell carcinoma	32 (34.8)	37 (33.3)	
Large cell carcinoma	2 (2.2)	4 (3.6)	
Other/not otherwise specified	8 (8.7)	5 (4.5)	
Treatment indication			1.000^b)^
Postoperative recurrence	40 (43.5)	48 (43.2)	
Advanced cancer	52 (56.5)	63 (56.8)	
ICI-related characteristics			
Types of ICI			0.732^c)^
Nivolumab	15 (16.3)	23 (20.7)	
Pembrolizumab	58 (63.0)	62 (55.9)	
Atezolizumab	15 (16.3)	19 (17.1)	
Durvalumab	4 (4.4)	7 (6.3)	
Checkpoint properties			0.735^b)^
PD-1 antibodies	73 (79.3)	85 (76.6)	
PD-L1 antibodies	19 (20.7)	26 (23.4)	
ICI treatment regimens			0.243^b)^
Monotherapy	61 (66.3)	83 (74.8)	
Combination with chemotherapy	31 (33.7)	28 (25.2)	
History of prior administration of anticancer agents			0.079^b)^
Yes	24 (26.1)	43 (38.7)	
No	68 (73.9)	68 (61.3)	
Brinkman index			0.036^b)^
<600	22 (23.9)	43 (38.7)	
≥600	70 (76.1)	68 (61.3)	
Comorbidities			
Diabetes mellitus			0.977^b)^
Yes	18 (19.6)	23 (20.7)	
No	74 (80.4)	88 (79.3)	
Heart failure			0.003^b)^
Yes	7 (7.6)	27 (24.3)	
No	85 (92.4)	84 (75.7)	
Obstructive ventilatory disorders			0.532^b)^
Yes	26 (28.3)	26 (23.4)	
No	66 (71.7)	85 (76.6)	
Autoimmune diseases			1.000^c)^
Yes	2 (2.2)	3 (2.7)	
No	90 (97.8)	108 (97.3)	
Regular use of strong opioids			0.508^b)^
Yes	11 (12.0)	18 (16.2)	
No	81 (88.0)	93 (83.8)	
Regular use of oral steroids			0.973^b)^
Yes	11 (12.0)	12 (10.8)	
No	81 (88.0)	99 (89.2)	
Regular use of inhaled steroids			0.517^c)^
Yes	6 (6.5)	4 (3.6)	
No	86 (93.5)	107 (96.4)	
Regular use of RAAS inhibitors			0.010^b)^
Yes	20 (21.7)	44 (39.6)	
No	72 (78.3)	67 (60.4)	
Aspartate aminotransferase (U/L)	20 [15–26]	21 [15–27]	0.470^a)^
Alanine aminotransferase (U/L)	16 [11–24]	14 [11-22.5]	0.600^a)^
Serum creatinine (mg/dL)	0.77 [0.63–0.88]	0.77 [0.65–0.94]	0.489^a)^
C-reactive protein (mg/L)	1.14 [0.30–3.65]	1.28 [0.39–5.28]	0.259^a)^
Number of ICI administrations	7 [3–16]	5 [2-16.5]	0.132^a)^

Discrete and continuous data are expressed as medians [interquartile ranges].

Categorical data are expressed as numbers (%)

*N* number, *irAEs* immune-related adverse events, *BMI* body mass index,

*ICI* immune checkpoint inhibitor, *PD-1* programmed cell death protein 1, *PD-L1* programmed death-ligand 1, *RAAS* renin–angiotensin–aldosterone system

a) Mann–Whitney U test

b) Pearson chi-squared test

c) Fisher’s exact test

### Cumulative incidence of irAEs

**Fig. 1:**
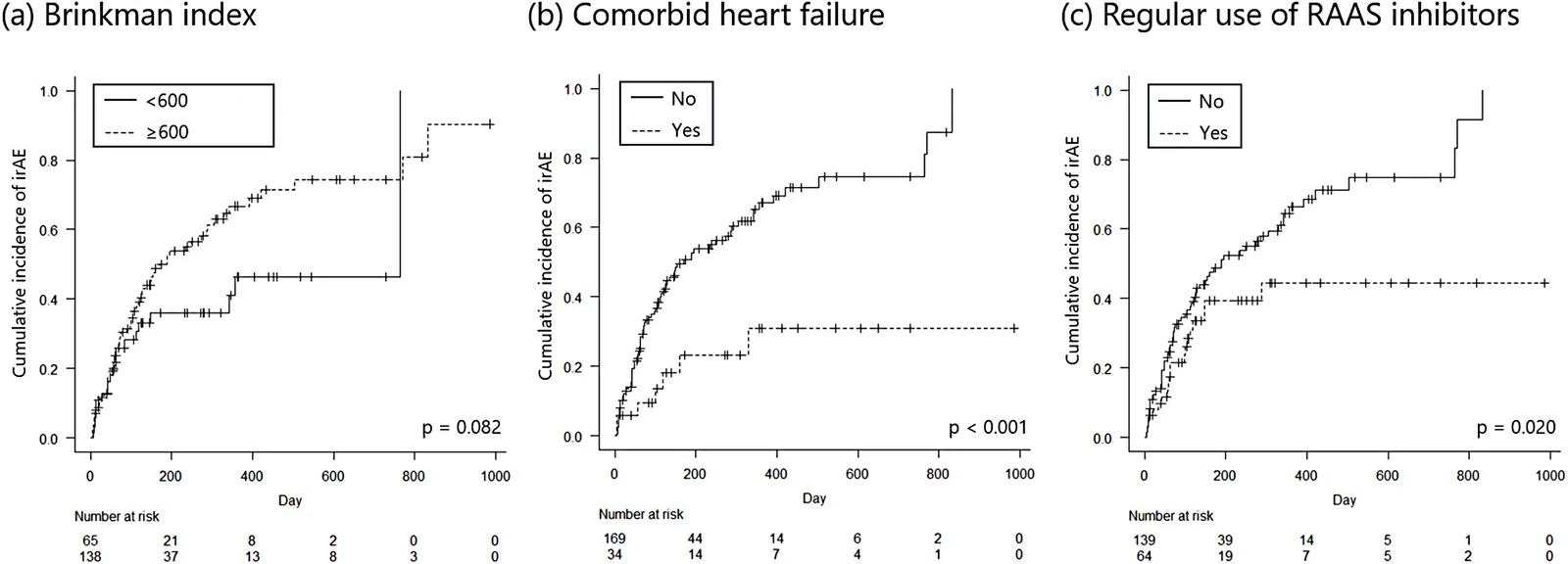
Cumulative incidence of irAEs. Cumulative incidence curves for immune‑related adverse events (irAEs) were estimated using a competing‑risk framework, in which death before irAE onset, disease progression, and discontinuation of immune checkpoint inhibitor (ICI) therapy were treated as competing events, while patients without irAEs were censored at the last follow‑up. (**a**) Cumulative incidence of irAEs stratified by Brinkman index (< 600 vs. ≥600). (**b**) Cumulative incidence of irAEs stratified by comorbid heart failure (yes vs. no). (**c**) Cumulative incidence of irAEs stratified by regular use of renin–angiotensin–aldosterone system (RAAS) inhibitors (yes vs. no). Numbers at risk for each group are shown below the x‑axis. Differences between groups were assessed using Gray’s test

Cumulative incidence curves were generated for the three variables that demonstrated significant differences in the univariate analysis—Brinkman index, comorbid heart failure, and regular use of RAAS inhibitors—and are shown in Fig. 1. Significant differences in the cumulative incidence of irAEs were observed according to comorbid heart failure (*p* < 0.001) and regular use of RAAS inhibitors (*p* = 0.020). In contrast, no significant difference in cumulative incidence was observed between Brinkman index categories.

### Time‑to‑event analysis

Time‑to‑event analysis using Cox proportional hazards regression was performed to identify factors associated with irAE development (Table 4). The variables included age, BMI, PD‑1 antibody use, combination chemotherapy, Brinkman index ≥ 600, comorbid heart failure, and C‑reactive protein levels. Regular use of RAAS inhibitors was excluded from the analysis because of potential multicollinearity with comorbid heart failure. In the multivariable Cox proportional hazards model, comorbid heart failure remained significantly associated with a reduced risk of irAE development after adjustment for covariates (HR, 0.299; 95% confidence interval (CI), 0.135–0.663; *p* = 0.003).

**Table 4:** Cox proportional hazards analysis for factors associated with irAE development (*N *= 203)

	HR	95% CI	*p*-value^a)^
Age	1.005	0.981–1.030	0.686
BMI	0.999	0.942–1.061	0.986
PD-1 antibodies	0.962	0.567–1.634	0.886
Combination with chemotherapy	1.544	0.945–2.521	0.083
Brinkman index (≥ 600)	1.383	0.844–2.269	0.199
Comorbid heart failure	0.299	0.135–0.663	0.003
C-reactive protein levels	1.023	0.982–1.065	0.277

Time-to-event was defined as the duration from initiation of immune checkpoint inhibitor therapy to the first immune-related adverse event. Patients without events were censored at the last follow-up. Disease progression, treatment discontinuation, or death before irAE onset were treated as censoring events

*irAEs*, immune-related adverse events; *HR*, hazard ratio; *CI*, confidence interval; *BMI*, body mass index; *PD-1*, programmed cell death protein 1

a) Wald test

### Competing risk analysis

In the overall cohort, 92 patients developed irAEs, whereas 57 experienced disease progression, 38 discontinued ICI therapy, and 16 died before irAE onset. To account for these competing events, a Fine–Gray competing risk model was applied using the same variables included in the time‑to‑event analysis (Table 5). In the competing risk model, comorbid heart failure remained significantly associated with a lower cumulative incidence of irAEs (SHR, 0.335; 95% CI, 0.147–0.766; *p* = 0.009).

**Table 5:** Fine–Gray subdistribution hazards analysis for factors associated with irAE development (*N* = 203)

	SHR	95% CI	*p*-value^a)^
Age	0.989	0.964–1.014	0.370
BMI	1.027	0.968–1.089	0.380
PD-1 antibodies	1.102	0.627–1.938	0.740
Combination with chemotherapy	1.266	0.795–2.016	0.320
Brinkman index (≥ 600)	1.573	0.939–2.634	0.085
Comorbid heart failure	0.335	0.147–0.766	0.009
C-reactive protein levels	0.973	0.929–1.018	0.240

In the overall cohort, 92 patients developed irAEs, while 57 experienced disease progression, 38 discontinued ICI therapy, and 16 died before irAE onset

*irAEs* immune-related adverse events, SHR subdistribution hazard ratio, *CI* confidence interval, *BMI* body mass index, *PD-1* programmed cell death protein 1

a) Wald test

### Sensitivity analysis incorporating regular use of RAAS inhibitors

To further evaluate whether regular use of RAAS inhibitors independently influenced irAE development, additional analyses were conducted using the same covariates as those included in the primary models: age, BMI, PD‑1 antibody use, combination chemotherapy, Brinkman index ≥ 600, regular RAAS inhibitor use, and C‑reactive protein levels. In the overall cohort (*N* = 203), regular use of RAAS inhibitors was significantly associated with a reduced risk of irAE development in both the time‑to‑event analysis (HR, 0.509; 95% CI, 0.299–0.867; *p* = 0.013) and the competing risk analysis (SHR, 0.502; 95% CI, 0.294–0.857; *p* = 0.012). Furthermore, the analyses were repeated after excluding patients with heart failure (*N* = 169; irAE events = 85). In this restricted cohort, although the hazard ratios remained numerically below 1.0, regular use of RAAS inhibitors was no longer significantly associated with irAE development in either the time‑to‑event analysis (HR, 0.638; 95% CI, 0.362–1.125; *p* = 0.120) or the competing risk analysis (SHR, 0.641; 95% CI, 0.363–1.130; *p* = 0.120).

## Discussion

This study elucidated irAE patterns within a single Japanese institutional cohort and suggested that comorbid heart failure may be associated with a lower incidence of irAEs. The advent of ICIs has substantially transformed therapeutic strategies for NSCLC and significantly improved patient outcomes [3–6]. Nevertheless, the development of irAEs remains a major clinical challenge that can impede treatment continuity [9]. Severe irAEs may result in treatment discontinuation, reduced efficacy, and deterioration in patients’ quality of life. Moreover, fatal cases directly attributable to irAEs have been reported [21–23]. Ensuring the safe and effective application of ICI-based therapy requires the accumulation of robust evidence to inform the prevention and early detection of irAEs.

In this study, we retrospectively evaluated the occurrence of irAEs in adult patients with NSCLC who received ICIs for the first time. Among the 203 patients included, the median age was 71 years, and 74.4% were men. Although activities of daily living were not formally assessed, all patients met the clinical criteria for ICI administration as determined by the attending physicians. Overall, 110 irAE events were documented in 92 patients, resulting in an incidence proportion of 45.3%. Reported irAE incidence proportions vary widely depending on the type of ICI and affected organ systems [7, 8, 10]. In NSCLC, a systematic review of 23 studies reported overall irAE rates of 64% for PD-1 inhibitors and 66% for PD-L1 inhibitors [24]. In a toxicity analysis of nivolumab in NSCLC, 40% of patients experienced irAEs of any grade, predominantly low-grade events [25]. The incidence proportion observed in this study was generally consistent with these previous findings.

In this survey, ILD was identified as the most common irAE, occurring in 23 patients (20.9%), representing a higher incidence than previously reported [26]. Suresh et al. suggested that the real-world incidence of ILD may be elevated due to ICI use outside clinical trial settings, and our findings support this assertion [5]. The median time to ILD onset was 70 d, consistent with prior reports [26]. When ILD was compared with other irAEs, no clear difference in onset timing was observed; the median time to onset for non‑ILD irAEs was 73 d. Among endocrine-related irAEs, thyroid dysfunction was the most prevalent, observed in 20 patients (18.2%). This finding aligns with earlier studies that reported thyroid dysfunction as the most frequent endocrine irAE [27]. Across all endocrine disorders, the median time to onset was 119 d, comparable to the reported range of 1.4–4.9 months [28, 29]. Gastrointestinal irAEs, such as colitis and severe diarrhea, occurred in 17 patients (15.5%) with a median onset of 98 d. Previous studies reported incidence rates of approximately 20–32% for colitis and 8% for severe diarrhea, similar to our findings [7]. However, previous reports indicated that onset typically occurs 4–6 weeks after treatment initiation, whereas in our study, onset was delayed [30]. This discrepancy may be due to racial differences in dietary habits or gut microbiota, although further investigation is warranted. The timing and incidence of other irAEs, including early-onset dermatologic disorders and hepatic dysfunction, were generally consistent with prior literature [31, 32]. Overall, this comprehensive analysis of irAEs in a Japanese NSCLC cohort demonstrated patterns similar to those observed in other ethnic populations. Although the difference was not significant, the irAE group received more rounds of ICIs than the non-irAE group. This observation aligns with the literature suggesting that irAE development may be associated with prolonged ICI therapy [33].

In this comprehensive study of irAEs, we examined factors potentially associated with their development. Comparative analysis between irAE and non-irAE groups revealed significant differences in the Brinkman index ≥ 600, comorbid heart failure, and regular use of RAAS inhibitors. These differences were also reflected in the cumulative incidence analysis, in which comorbid heart failure and regular use of RAAS inhibitors demonstrated significant separation of the cumulative incidence curves, whereas no significant difference was observed according to Brinkman index category. Building on these findings, multivariable time-to-event analysis was conducted using these categorical variables, excluding regular RAAS inhibitor use, along with established risk factors previously reported in the literature (age, BMI, PD-1 antibodies, combination with chemotherapy, and C-reactive protein levels) [11–13, 15]. The variance inflation factors for comorbid heart failure and regular use of RAAS inhibitors were 1.067 and 1.157, respectively. The phi coefficient calculated from a 2 × 2 contingency table for these two variables was 0.32, indicating a low likelihood of statistically meaningful multicollinearity. Nevertheless, the variable representing regular RAAS inhibitor use was excluded from the multivariable analysis because, from both clinical and descriptive statistical perspectives, potential multicollinearity with comorbid heart failure could not be fully ruled out.

The inverse association between comorbid heart failure and irAE development was evident in the cumulative incidence analysis and was consistently observed in both the Cox proportional hazards and Fine–Gray competing risk models. In patients with heart failure, blood concentrations of atrial natriuretic peptide and brain natriuretic peptide are known to be elevated. These hormones have been reported to act on macrophages and T cells, suppressing the production of proinflammatory cytokines such as interleukin-1β and tumor necrosis factor-α [34, 35]. Furthermore, experimental studies have indicated that atrial natriuretic peptide attenuates pulmonary fibrosis and inflammatory cell infiltration in animal models [36]. These effects may suppress excessive immune activation and potentially reduce the risk of irAEs. Considering the observational nature of this research, a direct association between heart failure and irAEs risk cannot be established. Although no cardiovascular irAEs were observed in the present study, a history of heart failure has been identified as a potential risk factor for cardiovascular disorders manifesting as irAEs [37]. Further mechanistic and longitudinal studies are warranted to clarify this potential relationship.

The observed association likely reflects not only disease‑related immunological alterations but also the effects of concomitant medications commonly used in this patient population. Among the 27 patients who had comorbid heart failure and did not develop irAEs, 18 (66.7%) were treated with RAAS inhibitors. Of the seven patients who developed irAEs, three (42.9%) received RAAS inhibitors. In the cumulative incidence analysis, regular use of RAAS inhibitors was associated with a significantly lower cumulative incidence of irAEs. Experimental studies using animal models and cell lines have shown that angiotensin-converting enzyme inhibitors and angiotensin II receptor blockers suppress inflammatory cytokine secretion and exert immunoregulatory effects [38, 39]. RAAS inhibitors have been suggested to exert beneficial effects on various systemic pathological conditions through their immunomodulatory properties [40]. Furthermore, several clinical studies have examined the impact of RAAS inhibitors on ICI-based immunotherapy, reporting improved treatment outcomes [41–43]. However, to date, no study has specifically examined the association between RAAS inhibitors and the incidence of irAEs. Based on previous evidence and our findings, RAAS inhibitors may mitigate irAEs development via immunomodulatory mechanisms. In our sensitivity analyses incorporating regular RAAS inhibitor use as an independent covariate, regular use of RAAS inhibitors was associated with a reduced risk of irAEs in the overall cohort; however, this association was no longer observed in the subgroup analysis excluding patients with heart failure. Although the influence of the limited sample size cannot be fully excluded, this pattern suggests that the apparent protective effect associated with RAAS inhibitors may not solely reflect an independent pharmacologic action but may instead be partly attributable to the suppression of excessive immune activation associated with comorbid heart failure.

From a clinical perspective, the inverse association between heart failure and irAE development should be interpreted cautiously. Patients with heart failure often have reduced physiological reserve, and the occurrence of severe irAEs in this population may result in more serious clinical consequences than in patients without cardiac comorbidities. Therefore, these findings do not justify less intensive irAE surveillance in patients with heart failure. Clinicians should recognize that heart failure and its associated treatments may shift the immunological milieu toward a less proinflammatory state while still maintaining a high index of suspicion for both immune‑mediated and non‑immune‑mediated adverse events. In practical terms, risk stratification should account for both the potential attenuation of systemic inflammatory responses and the increased vulnerability of patients with heart failure to treatment‑related decompensation. Further mechanistic studies are warranted to determine whether heart failure represents a true modifier of irAE susceptibility or a surrogate marker for unmeasured clinical characteristics influencing immune activation.

A previous meta‑analysis reported an association between smoking and an increased risk of irAEs in NSCLC [13]. Smoking exerts multifaceted immunological effects, including chronic lung inflammation, aberrant cytokine secretion, and immune cell activation [44, 45]. Moreover, smoking-induced alterations in the immune microenvironments may interact with ICI mechanisms; for example, smoking has been linked to T-cell exhaustion and modulation of PD-1/PD-L1 pathway expression, which may influence irAE susceptibility [46, 47]. In our cohort, patients with a Brinkman index ≥ 600 showed a higher proportion of irAEs in the two‑group comparison; however, this association was not reproduced in the cumulative incidence analysis or in the Cox and Fine–Gray models, and the Brinkman index was not independently associated with irAE development. Among the 23 patients who developed ILD, 18 (78.3%) had a Brinkman index ≥ 600. Furthermore, in the subgroup analysis excluding patients with ILD (*N* = 180), no association was observed between Brinkman index and irAE development (data not shown). These findings suggest that the initial signal observed in the univariate analysis likely reflects the contribution of smoking to pulmonary irAEs rather than a systemic predisposition to irAEs across multiple organ systems. Thus, although heavy smoking may increase susceptibility to lung‑related irAEs, particularly ILD, it does not appear to represent an independent risk factor for overall irAE development in patients receiving ICIs. Nevertheless, further investigation is warranted to clarify the relationship between smoking and systemic irAEs.

This study has some limitations. First, the retrospective single‑center design limits the generalizability of the findings. Retrospective data collection may have resulted in incomplete capture or misclassification of irAEs and clinical variables, while institutional differences in diagnostic thresholds and irAE management strategies may limit extrapolation of the findings to other settings. In addition, the relatively small sample size raises the possibility of selection bias. Second, accurately ascertaining patient characteristics, including performance status, therapeutic response to ICIs, PD-L1 expression status, and disease severity, was challenging. Laboratory parameters previously reported to be associated with irAE risk, such as the neutrophil-to-lymphocyte ratio, were excluded from the analysis. These clinical variables may influence the development of irAEs and could constitute confounding factors. Third, as irAEs were analyzed in aggregate, caution is warranted when extrapolating these findings to specific irAE types. Clinical decisions should be supported by both the present results and prior studies focusing on individual irAE profiles. Finally, although heart failure emerged as a potential modifier of irAE development, detailed phenotypic information—such as heart failure with reduced versus preserved ejection fraction, underlying etiology, disease severity, and biomarker profiles—was not available. Because these phenotypes differ in inflammatory activity, immune regulation, and susceptibility to treatment‑related stress, the absence of such data limits the ability to determine how specific heart failure subtypes may influence irAE susceptibility. Future studies should comprehensively assess patient backgrounds, including clinical history and pathological conditions, to better elucidate the multifactorial nature of irAE development.

## Conclusions

Overall, this study provides a comprehensive evaluation of irAEs associated with ICI therapy in Japanese patients with NSCLC and suggests that comorbid heart failure may be associated with a lower incidence of irAEs in time‑to‑event analyses. These findings indicate that baseline cardiac conditions may influence susceptibility to immune‑related toxicity and underscore the importance of incorporating comorbidity profiles into pretreatment assessment and individualized irAE monitoring strategies. Such approaches may help optimize the safety of immunotherapy in patients with diverse clinical backgrounds.

## Data Availability

The data cannot be shared according to the regulations of the ethics committee of the Tohoku Medical and Pharmaceutical University Hospital.

## Abbreviations

BMI: Body mass index
CI: Confidence interval
CTCAE: Common Terminology Criteria for Adverse Events
HR: Hazard ratio
ICI: Immune checkpoint inhibitor
ILD: Interstitial lung disease
IQR: Interquartile range
irAE: Immune-related adverse event
NSCLC: Non-small cell lung cancer
PD-1: Programmed cell death protein 1
PD-L1: Programmed death-ligand 1
RAAS: Renin-angiotensin-aldosterone system
SHR: Subdistribution hazard ratio
